# Association of Long-term Use of Low-Dose Aspirin as Chemoprevention With Risk of Lung Cancer

**DOI:** 10.1001/jamanetworkopen.2019.0185

**Published:** 2019-03-01

**Authors:** Shinhee Ye, Myeongjee Lee, Dongheon Lee, Eun-Hee Ha, Eun Mi Chun

**Affiliations:** 1 Department of Occupational and Environmental Medicine, School of Medicine, Ewha Womans University, Seoul, Republic of Korea; 2 Occupational Safety and Health Research Institute, Korea Occupational Safety and Health Agency, Incheon, Republic of Korea; 3 Department of Statistics, Williams College, Williamstown, Massachusetts; 4 Division of Pulmonology and Critical Care Medicine, Department of Internal Medicine, School of Medicine, Ewha Womans University, Seoul, Republic of Korea

## Abstract

**Importance:**

Low-dose aspirin use for chemoprevention of lung cancer risk remains controversial.

**Objectives:**

To investigate the association between low-dose aspirin use and lung cancer risk, and to identify specific subgroups that may derive the most benefit from low-dose aspirin use.

**Design, Setting, and Participants:**

This nationwide, retrospective, cohort study used data from the Korean National Health Information Database from 2002 to 2015. Data analyses were performed from October 2016 to December 2018. Eligible participants (n = 12 969 400) were people aged 40 to 84 years who had undergone national health screening between 2009 and 2010 and had no history of lung cancer between 2006 and 2010 and no standard-dose aspirin use for 6 months between 2002 and 2010.

**Main Outcomes and Measures:**

The duration of low-dose aspirin use between January 2002 and December 2010 was calculated for each participant. Lung cancer was defined as the first recorded diagnosis of lung cancer—using *International Statistical Classification of Diseases and Related Health Problems, Tenth Revision* codes and expanding benefit coverage—between January 2011 and December 2015.

**Results:**

A total of 63 040 participants with a mean (SD) age of 66.4 (9.3) years received a diagnosis of lung cancer. Of these, 45 156 (71.6%) were men. The incidence rate of lung cancer was 98.8 per 100 000 person-years. The duration of low-dose aspirin use was none for 10 987 417 participants (84.7%), 1 to 2 years for 750 992 participants (5.8%), 3 to 4 years for 506 945 participants (3.9%), 5 to 6 years for 371 062 participants (2.9%), 7 to 8 years for 240 528 participants (1.9%), and 9 years for 112 456 participants (0.9%). Compared with no aspirin use, 5 to 6 years (adjusted hazard ratio, 0.96 [95% CI, 0.92-0.99]), 7 to 8 years (adjusted hazard ratio, 0.94 [95% CI, 0.90-0.99]), and 9 years (adjusted hazard ratio, 0.89 [95% CI, 0.84-0.94]) of aspirin use were significantly associated with reduced lung cancer risk. After stratified analysis, a significant reduction of lung cancer risk was observed among people aged 65 years or older and among people without diabetes.

**Conclusions and Relevance:**

Although the use of low-dose aspirin for more than 5 years was associated with decreased risk of lung cancer, particularly among elderly participants and among people without diabetes, the observed effect size was quite modest. Future prospective studies are needed to determine whether there is a causal association.

## Key Points

**Question:**

Is long-term use of low-dose aspirin associated with reduced risk of lung cancer, and if so, which populations may derive the greatest benefit?

**Findings:**

This cohort study using data from 12 969 400 participants in the Korean National Health Information Database found that the use of low-dose aspirin for more than 5 years was associated with a modest risk reduction of incident lung cancer, with the strongest association observed among elderly participants and among people without diabetes.

**Meaning:**

Intake of low-dose aspirin for more than 5 years may reduce the risk of incident lung cancer, particularly among the elderly and among people without diabetes.

## Teaser

This nationwide, population-based cohort study uses data from the Korean National Health Information Database to assess whether an association exists between low-dose aspirin use and lung cancer risk and to identify specific subgroups that may benefit most from low-dose aspirin use.

## Introduction

Lung cancer was the most commonly diagnosed cancer (1.8 million cases; 12.9% of all cancer cases) and the most common cause of cancer death (1.6 million deaths; 19.4% of all cancer deaths) in 2012 worldwide.^1^ The risk of lung cancer remains elevated after smoking cessation,^2^ and 15% of men and 53% of women with lung cancer are lifelong nonsmokers.^3^ Given this significant health burden, additional approaches for preventing lung cancer have been suggested, including regular aspirin use. Previous studies of aspirin use in patients with cardiovascular disease have suggested that low-dose aspirin use is beneficial in reducing the risk of cancer in a duration-dependent manner.^4,5^

The antineoplastic effect of aspirin may be associated with the inhibition of cyclooxygenase-1 (COX-1)–mediated platelet activation.^6^ Inhibition of arterial thrombosis by aspirin impedes the development and progression of certain cancers.^7^ Cyclooxygenase-2 is known to be involved in the inflammatory process, and inhibition of COX-2–derived prostaglandin E_2_ formation by aspirin induces inhibition of cell proliferation and reduction of angiogenesis and immunomodulation through the production of lymphocytes in the peripheral blood.^8^ Cyclooxygenase-2 is expressed at high levels in human lung cancer tissue, especially in adenocarcinoma.^9^

According to the results of 2 meta-analyses, despite significant findings on aspirin use and lung cancer risk among case-control studies, the chemopreventive effects of aspirin on lung cancer risk in cohort studies were controversial.^10,11^ A pooled analysis of randomized trials with long-term follow-up showed that daily aspirin use reduced death due to lung cancer.^12^ Thus, the association between low-dose aspirin use and the risk reduction of lung cancer, the appropriate duration of aspirin use for chemoprevention, and the specific subgroups expected to benefit more from low-dose aspirin use are still inconclusive.

The present study aimed to examine the association between long-term use of low-dose aspirin and the risk of incident lung cancer through a large-scale, nationwide, population-based, cohort study. This study also aimed to investigate the appropriate duration of aspirin intake for chemoprevention and to identify specific subgroups that may benefit more from low-dose aspirin use as chemoprevention.

## Methods

### Data Sources

**Figure 1.**
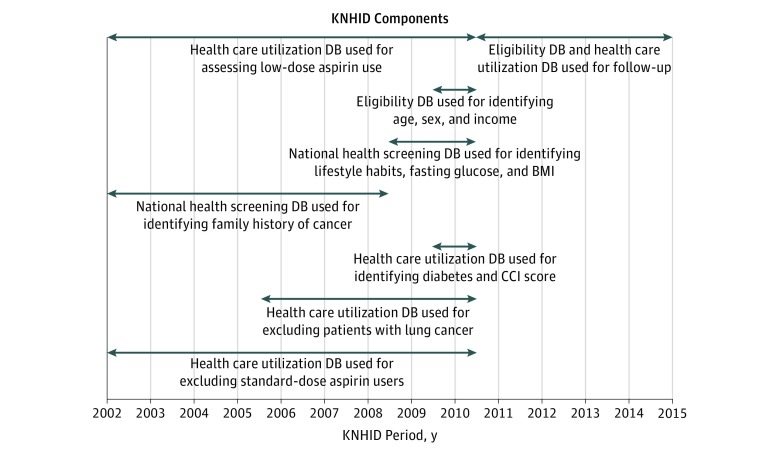
Schematic Representation of the Korean National Health Information Database (KNHID) Periods Used BMI indicates body mass index; CCI, Charlson comorbidity index. Arrows indicate the years in which data from specific databases (DBs) were used in the present study.

This nationwide, retrospective, cohort study used data from the Korean National Health Information Database (KNHID) collected between January 1, 2002, and December 31, 2015. Data analysis was performed from October 2016 to December 2018, and preliminary data analysis using a sample cohort was conducted from July 2015 to September 2016. The KNHID includes information about participants who visited hospitals under the Korean National Health Insurance Service program, which covers all legal residents of the Republic of Korea.^13^ The KNHID consists of 5 databases (eligibility database, national health screening database, health care utilization database, long-term care insurance database, and medical institution database) and includes public data on health care use, health screening, sociodemographic variables, and mortality.^13,14^ The types and duration of component databases used in this study are illustrated in Figure 1. The present study was approved by the Institutional Review Board of Ewha Womans University Medical Center and the National Health Insurance Service, which also waived the need for obtaining informed patient consent because the KNHID was constructed after anonymization according to strict confidentiality guidelines. We followed the Strengthening the Reporting of Observational Studies in Epidemiology (STROBE) reporting guideline^15^ for reporting the results of the present study.

### Study Population

**Figure 2.**
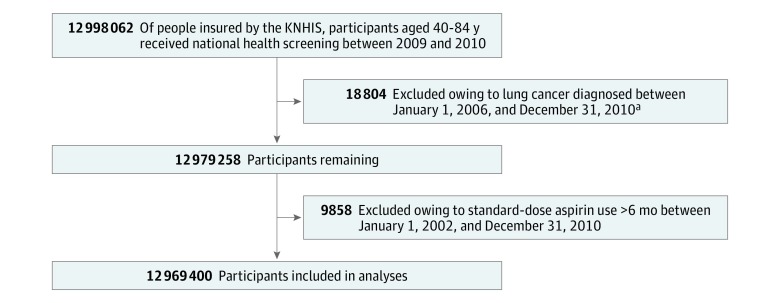
Flowchart of the Study Population ^a^*International Statistical Classification of Diseases and Related Health Problems, Tenth Revision* (*ICD-10*) codes C33 (malignant neoplasm of trachea) and C34 (malignant neoplasm of bronchus and lung) were used to identify patients with lung cancer, using the principal diagnosis or first additional diagnosis. The code for expanding benefit coverage was also used to increase accuracy of lung cancer diagnosis. KNHIS represents Korean National Health Insurance Service.

The study included 12 998 062 participants, aged 40 to 84 years during 2010, who underwent national health screening between January 1, 2009, and December 31, 2010. Of them, 18 804 participants were excluded because they had received a diagnosis of lung cancer between January 1, 2006, and December 31, 2010. In addition, 9858 participants were excluded because they were prescribed standard doses (≥325 mg)^10^ of aspirin for more than 6 months between January 1, 2002, and December 31, 2010 (Figure 2; eTable 1 in the Supplement).

### Definition of Lung Cancer

Using the heath care utilization database, participants with lung cancer during follow-up between January 1, 2011, and December 31, 2015, were defined as those with *International Statistical Classification of Diseases and Related Health Problems, Tenth Revision* (*ICD-10*) codes C33.x (malignant neoplasm of trachea) or C34.x (malignant neoplasm of bronchus and lung), in the principal or first additional diagnosis with the code for expanding benefit coverage, which is a Korean policy that commenced in September 2005 (eTable 2 in the Supplement). This policy reduces the medical expenses of patients with catastrophic illnesses, such as cancer or cardiovascular disease or severe burn injury.

### Assessment of Aspirin Use

For the exposure assessment, we used the health care utilization database collected between January 1, 2002, and December 31, 2010. In the present study, low-dose aspirin was defined as a dose of 100 mg or less. The prescription data from eligible participants showed that 94.7% of the prescriptions instructed taking 1 tablet at a time, and 94.6% of the prescriptions directed taking aspirin once daily (eTable 3 in the Supplement).

Participants who were prescribed low-dose aspirin for at least 104 days were defined as low-dose aspirin users in each year, which means that a person took at least 2 aspirin tablets per week during a year.^16,17^ Those who were prescribed aspirin for less than 104 days in a year were defined as nonusers of low-dose aspirin in each year. Nonusers of low-dose aspirin also included those who had never used low-dose aspirin. The duration of low-dose aspirin use was then categorized into 2-year ranges: none, 1 to 2, 3 to 4, 5 to 6, 7 to 8, and 9 years (Table 1).

**Table 1.** Representative Schematic for Assessment of Duration of Low-Dose Aspirin Use

Low-Dose Aspirin Use, y	Periods of Low-Dose Aspirin Use^a^								
	2002	2003	2004	2005	2006	2007	2008	2009	2010
0	No	No	No	No	No	No	No	No	No
1-2	No	No	No	No	No	No	Yes	No	No
3-4	No	No	No	No	No	Yes	Yes	Yes	Yes
5-6	No	No	No	Yes	No	Yes	Yes	Yes	Yes
7-8	No	Yes	Yes	Yes	Yes	Yes	Yes	Yes	Yes
9	Yes	Yes	Yes	Yes	Yes	Yes	Yes	Yes	Yes

^a^ No indicates a year in which participants did not use low-dose aspirin; yes, a year in which participants used low-dose aspirin.

### Smoking and Other Covariates

For information on health behaviors (smoking status, alcohol consumed, and physical activity), physical examination (body mass index [BMI]; calculated as weight in kilograms divided by height in meters squared), and fasting serum glucose levels, we used the most recent national health screening results from the national health screening database collected between January 1, 2009, and December 31, 2010. Data on diagnoses of type 1 and type 2 diabetes were collected using *ICD-10* codes (E10.x and E11.x) from the 2010 health care utilization database. Participants reported smoking status as nonsmokers, past smokers, or current smokers. Depending on pack-years of smoking, the past smokers and current smokers were reclassified into nonsmokers (0 pack-years of smoking) or smokers of less or more than 30 pack-years of smoking. Alcohol consumption was categorized as no consumption or as 1 to 7, 8 to 14, and more than 15 units of alcohol consumed in a week. The frequency of moderate or vigorous exercise per week was categorized as no exercise, 1 to 4 times per week, and more than 5 times per week. Participant BMI was categorized as less than 18.5 (underweight), 18.5 to 22.9 (normal weight), 23.0 to 24.9 (overweight), 25.0 to 29.9 (obese), and 30 or greater (morbidly obese). Patients with diabetes were defined by their fasting serum glucose level (≥126 mg/dL; to convert to millimoles per liter, multiply by 0.0555) or by a diagnosis of type 1 or type 2 diabetes.

Information regarding family history of cancer was collected from the national health screening database between January 1, 2002, and December 31, 2008. The Charlson comorbidity index score was calculated using *ICD-10* codes obtained from the health care utilization database for 2010.^18^ Information on variables of age (years), sex, income (quintile), and residential area (metropolitan, city, and rural areas) was obtained from the eligibility database for 2010.

### Statistical Analysis

All participants were followed up from baseline (January 1, 2011) until the date of lung cancer diagnosis (event of interest), death by any reason (competing event), censoring due to loss of health insurance eligibility (eg, loss of nationality or moving overseas, or nonpayment of insurance cost), or the end of the follow-up period (December 31, 2015), whichever occurred first (eTable 4 in the Supplement). The follow-up duration in days was used as the time scale.

Competing risk analysis (Fine and Gray model)^19^ was performed with consideration of death as a competing event. This analysis was conducted to reduce the bias from competing events that either hinder or alter the chance of an event of interest occurring.^20^

For the multivariate analysis, the association between duration of low-dose aspirin use and the risk of incident lung cancer was evaluated using 2 models, an age- and sex-adjusted model and a fully adjusted model. The fully adjusted model used possible confounders of age, sex, income, residential area, pack-years of smoking, quantity of alcohol consumed, frequency of moderate or vigorous exercise, BMI, history of diabetes, family history of cancer, and Charlson comorbidity index score; age was a continuous variable and other variables were categorical variables, as shown in Table 2. Missing values in the covariates were included in the model as a missing indicator category. A trend test was performed with the duration of low-dose aspirin use treated as a numeric variable in the models.

**Table 2.** Characteristics of Study Participants at Baseline by Duration of Low-Dose Aspirin Use^a^

Characteristic	No. (%) of Participants by Duration of Low-Dose Aspirin Use					
	None (n = 10 987 417)^b^	1-2 y (n = 750 992)	3-4 y (n = 506 945)	5-6 y (n = 371 062)	7-8 y (n = 240 528)	9 y (n = 112 456)
Sex						
Men	5 340 594 (48.6)	367 882 (49.0)	252 254 (49.8)	184 999 (49.9)	120 361 (50.0)	58 755 (52.3)
Women	5 646 913 (51.4)	383 110 (51.0)	254 691 (50.2)	186 063 (50.1)	120 167 (50.0)	53 701 (47.8)
Age, y						
40-49	4 249 519 (38.7)	72 283 (9.6)	35 948 (7.1)	17 956 (4.8)	7402 (3.1)	2 033 (1.8)
50-59	3 714 239 (33.8)	218 497 (29.1)	137 387 (27.1)	89 198 (24.0)	48 520 (20.2)	18 231 (16.2)
60-69	1 945 112 (17.7)	252 270 (33.6)	179 501 (35.4)	138 431 (37.3)	91 433 (38.0)	42 525 (37.8)
70-79	953 159 (8.7)	180 507 (24.0)	133 967 (26.4)	109 207 (29.4)	80 835 (33.6)	42 909 (38.2)
80-84	125 388 (1.1)	27 435 (3.7)	20 142 (4.0)	16 270 (4.4)	12 338 (5.1)	6758 (6.0)
Income, quintile^c^						
1-4	1 608 109 (14.6)	111 661 (14.9)	75 130 (14.8)	54 584 (14.7)	34 265 (14.3)	15 247 (13.6)
5-8	1 499 008 (13.6)	93 962 (12.5)	62 179 (12.3)	43 473 (11.7)	27 147 (11.3)	11 880 (10.6)
9-12	1 764 779 (16.1)	117 907 (15.7)	78 144 (15.4)	55 412 (14.9)	34 720 (14.4)	15 233 (13.6)
13-16	2 388 306 (21.7)	165 441 (22.0)	111 452 (22.0)	81 723 (22.0)	52 730 (21.9)	24 345 (21.7)
17-20	3 467 266 (31.6)	241 587 (32.2)	167 128 (33.0)	126 176 (34.0)	86 230 (35.9)	43 355 (38.6)
Unknown	259 949 (2.4)	20 434 (2.7)	12 912 (2.6)	9694 (2.6)	5436 (2.3)	2 396 (2.1)
Residential area^d^						
Metropolitan	4 869 242 (44.3)	315 100 (42.0)	213 711 (42.2)	160 954 (43.4)	108 149 (45.0)	53 534 (47.6)
Urban	4 883 189 (44.4)	322 953 (43.0)	218 074 (43.0)	158 833 (42.8)	103 122 (42.9)	47 014 (41.8)
Rural area	1 234 974 (11.2)	112 939 (15.0)	75 158 (14.8)	51 275 (13.8)	29 257 (12.2)	11 908 (10.6)
Unknown	12 (0.0)	0	2 (0.0)	0	0	0
Smoking status						
Nonsmoker	7 014 542 (63.8)	494 656 (65.9)	334 855 (66.1)	247 622 (66.7)	162 195 (67.4)	74 840 (66.6)
Past smoker	1 645 484 (15.0)	132 986 (17.7)	94 104 (18.6)	70 183 (18.9)	47 057 (19.6)	24 014 (21.4)
Current smoker	2 327 391 (21.2)	123 350 (16.4)	77 986 (15.4)	53 257 (14.4)	31 276 (13.0)	13 602 (12.1)
Pack-years of smoking^e^						
Nonsmoker	7 019 085 (63.9)	494 902 (65.9)	334 993 (66.1)	247 732 (66.8)	162 264 (67.5)	74 862 (66.6)
<30	2 978 882 (27.1)	162 027 (21.6)	108 483 (21.4)	76 955 (20.7)	48 744 (20.3)	23 291 (20.7)
≥30	876 676 (8.0)	87 214 (11.6)	58 761 (11.6)	43 024 (11.6)	27 300 (11.4)	13 169 (11.7)
Unknown	112 774 (1.0)	6849 (0.9)	4708 (0.9)	3351 (0.9)	2220 (0.9)	1134 (1.0)
Alcohol consumption, units/wk^f^						
0	6 287 389 (57.2)	490 769 (65.4)	331 507 (65.4)	245 586 (66.2)	162 350 (67.5)	77 134 (68.6)
1-7	1 977 613 (18.0)	102 430 (13.6)	69 100 (13.6)	50 206 (13.5)	32 700 (13.6)	15 395 (13.7)
8-14	992 168 (9.0)	54 230 (7.2)	37 393 (7.4)	27 102 (7.3)	16 872 (7.0)	7551 (6.7)
≥15	1 616 586 (14.7)	95 575 (12.7)	63 504 (12.5)	44 332 (12.0)	26 000 (10.8)	11 153 (9.9)
Unknown	113 661 (1.0)	7988 (1.1)	5441 (1.1)	3836 (1.0)	2606 (1.1)	1223 (1.1)
Moderate or vigorous exercise, times/wk^g^						
None	5 394 811 (49.1)	400 027 (53.3)	267 665 (52.8)	195 978 (52.8)	126 280 (52.5)	59 149 (52.6)
1-4	3 422 854 (31.2)	190 315 (25.3)	127 035 (25.1)	91 145 (24.6)	58 128 (24.2)	26 504 (23.6)
≥5	2 103 812 (19.2)	155 583 (20.7)	108 746 (21.5)	81 439 (22.0)	54 521 (22.7)	26 015 (23.1)
Unknown	65 940 (0.6)	5067 (0.7)	3499 (0.7)	2500 (0.7)	1 599 (0.7)	788 (0.7)
BMI						
<18.5	274 219 (2.5)	11 064 (1.5)	6153 (1.2)	3989 (1.1)	2266 (0.9)	1118 (1.0)
18.5-22.9	4202 202 (38.3)	196 211 (26.1)	119 375 (23.6)	82 536 (22.2)	51 788 (21.5)	23 462 (20.9)
23-24.9	2902 397 (26.4)	198 049 (26.4)	132 060 (26.1)	96 659 (26.1)	62 345 (25.9)	29 462 (26.2)
25-29.9	3277 695 (29.8)	302 660 (40.3)	216 725 (42.8)	162 662 (43.8)	107 378 (44.6)	50 589 (45.0)
≥30	328 169 (3.0)	42 575 (5.7)	32 338 (6.4)	25 039 (6.8)	16 637 (6.9)	7785 (6.9)
Unknown	2735 (0.0)	433 (0.1)	294 (0.1)	177 (0.1)	114 (0.1)	40 (0.0)
History of diabetes^h^						
No	10 161 443 (92.5)	574 210 (76.5)	375 459 (74.1)	269 647 (72.7)	172 259 (71.6)	80 480 (71.6)
Yes	825 974 (7.5)	176 782 (23.5)	131 486 (25.9)	101 415 (27.3)	68 269 (28.4)	31 976 (28.4)
Family history of cancer						
No	6 519 478 (59.3)	492 829 (65.6)	340 753 (67.2)	251 828 (67.9)	164 430 (68.4)	76 323 (67.9)
Yes	1 805 635 (16.4)	114 278 (15.2)	75 762 (14.9)	54 059 (14.6)	33 834 (14.1)	15 403 (13.7)
Unknown	2 662 304 (24.2)	143 885 (19.2)	90 430 (17.8)	65 175 (17.6)	42 264 (17.6)	20 730 (18.4)
CCI score^i^						
0	3 902 241 (35.5)	54 735 (7.3)	26 895 (5.3)	13 419 (3.6)	5584 (2.3)	1593 (1.4)
1	3 472 931 (31.6)	162 231 (21.6)	100 471 (19.8)	64 012 (17.3)	34 414 (14.3)	13 026 (11.6)
2	1 918 188 (17.5)	191 838 (25.5)	132 090 (26.1)	98 567 (26.6)	63 129 (26.3)	29 183 (26.0)
3	1 059 862 (9.7)	164 324 (21.9)	117 806 (23.2)	91 756 (24.7)	63 916 (26.6)	32 212 (28.6)
≥4	634 195 (5.8)	177 864 (23.7)	129 683 (25.6)	103 308 (27.8)	73 485 (30.6)	36 442 (32.4)

Abbreviations: BMI, body mass index (calculated as weight in kilograms divided by height in meters squared); CCI, Charlson comorbidity index.

^a^ Duration of low-dose aspirin (≤100 mg) use calculated between January 1, 2002, and December 31, 2010; people prescribed low-dose aspirin for 104 days or longer during a year were defined as low-dose aspirin users in that year.

^b^ People who had never used low-dose aspirin.

^c^ Quintiles based on the population size of each national health insurance–determined premium level.

^d^ Classified on the basis of participant residential address.

^e^ If participants responded as a past or current smoker on the general health screening questionnaire, pack-years of smoking were calculated by multiplying years of cigarettes smoked with number of cigarettes typically smoked in a day. Those who smoked 0 pack-years were reclassified as nonsmokers.

^f^ Calculated based on the questionnaire of national health screening; type of alcohol was not considered.

^g^ Calculated based on the general health screening questionnaire.

^h^ Diabetes defined by fasting serum glucose levels (≥126 mg/dL; to convert to millimoles per liter, multiply by 0.0555) or type 1 and 2 diabetes diagnosis using *International Statistical Classification of Diseases and Related Health Problems, Tenth Revision* (*ICD-10*) codes E10.x and E11.x.

^i^ Calculated using *ICD-10* codes from the health care utilization database.

Stratified analyses examined differences in associations between long-term use of low-dose aspirin and lung cancer risk, defined by sex (men or women), age at baseline (<65 years or ≥65 years), BMI (<25 or ≥25), history of diabetes (yes or no), and pack-years of smoking (nonsmoker, <30 pack-years, or ≥30 pack-years).

We conducted 8 sensitivity analyses as follows. (1) A conventional survival analysis (Cox proportional hazards model)^21^ was conducted to compare previous cohort studies.^22,23^ (2) We analyzed the association in the 1:1 propensity score-matched cohort between aspirin users and nonusers. (3) Because some participants were taking aspirin during the follow-up period (eTable 5 in the Supplement), we analyzed the association between incident lung cancer and the duration of aspirin use from 2002 to the year in which the event occurred or the follow-up was censored. (4) Because 20% of the family history data were missing, we conducted an analysis in the fully adjusted model that excluded the family history variable. (5) Several operational definitions for the assessment of low-dose aspirin intake between January 2002 and December 2010 were used for the fully adjusted model. First, participants who were prescribed low-dose aspirin for at least 156, 208, or 260 days in each year were defined as a person who took at least 3, 4, or 5 aspirin tablets per week, respectively. Second, the total days of low-dose aspirin prescribed was used. (6) In the fully adjusted model, we used smoking status instead of pack-years of smoking for the adjustment. (7) We analyzed the fully adjusted model after excluding cases that occurred during the first year of follow-up. (8) We conducted a subgroup analysis with a combined 7 to 9 years of low-dose aspirin use group.

All statistical analyses were conducted using SAS, version 9.4 (SAS Institute Inc). The criterion for statistical significance was a 2-sided *P* < .05.

## Results

In total, 12 969 400 participants were enrolled in this study (Figure 2). The characteristics of the excluded and included participants are shown in eTable 1 in the Supplement.

The duration of low-dose aspirin use was none for 10 987 417 participants (84.7%), 1 to 2 years for 750 992 participants (5.8%), 3 to 4 years for 506 945 participants (3.9%), 5 to 6 years for 371 062 participants (2.9%), 7 to 8 years for 240 528 participants (1.9%), and 9 years for 112 456 participants (0.9%). Longer duration of low-dose aspirin use was associated with male sex, older age, high income, high BMI, history of diabetes, and high Charlson comorbidity index score (Table 2).

Total person-years of follow-up were 63 787 432.9 person-years for the lung cancer outcome. Among the 12 969 400 participants, 63 040 (0.5%) incident cases of lung cancer were detected. The incidence rate of the event was 98.8 per 100 000 person-years. The mean (SD) age of the patients with lung cancer was 66.4 (9.3) years, and 45 156 (71.6%) were men and 17 884 (28.4%) were women (eTable 6 in the Supplement).

**Figure 3.**
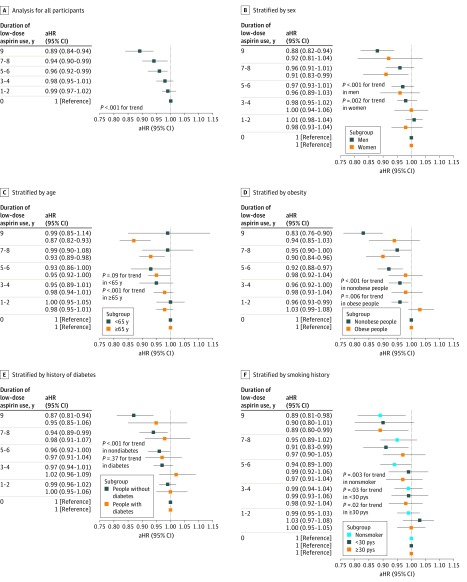
Association Between Long-term Use of Low-Dose Aspirin and Reduced Risk of Incident Lung Cancer This figure shows that the duration of low-dose aspirin use should be more than 5 years to achieve a significant association between low-dose aspirin use and risk of incident lung cancer, and that except for younger people and patients with diabetes, the risk of incident lung cancer decreases with longer duration of low-dose aspirin use. Error bars indicate 95% CIs; aHR, adjusted hazard ratio; pys, pack-years.

The results of the fully adjusted model are presented in Figure 3. In all participants, compared with no use, low-dose aspirin intake for more than 5 years was significantly associated with reduced lung cancer risk in a duration-dependent manner (adjusted hazard ratio [aHR]: 5-6 years’ use, 0.96 [95% CI, 0.92-0.99]; 7-8 years’ use, 0.94 [95% CI, 0.90-0.99]; and 9 years’ use, 0.89 [95% CI, 0.84-0.94]; *P* < .001 for trend).

Long-term use of low-dose aspirin was significantly associated with reduced risk of lung cancer among both men (aHR: 5-6 years’ use, 0.97 [95% CI, 0.93-1.01]; 7-8 years’ use, 0.96 [95% CI, 0.91-1.01]; 9 years’ use, 0.88 [95% CI, 0.82-0.94]; *P* < .001 for trend) and women (aHR [95% CI, 95% CI]: 5-6 years’ use, 0.96 [95% CI, 0.89-1.03]; 7-8 years’ use, 0.91 [95% CI, 0.83-0.99]; 9 years’ use, 0.92 [95% CI, 0.81-1.04]; *P* = .002 for trend). Long-term use of low-dose aspirin was also significantly associated with reduced risk of lung cancer among both nonobese (aHR: 5-6 years’ use, 0.92 [95% CI, 0.88-0.97]; 7-8 years’ use, 0.95 [95% CI, 0.90-1.00]; 9 years’ use, 0.83 [95% CI, 0.76-0.90]; *P* < .001 for trend) and obese participants (aHR: 5-6 years’ use, 0.98 [95% CI, 0.92-1.04]; 7-8 years’ use, 0.90 [95% CI, 0.84-0.96]; 9 years’ use, 0.94 [95% CI, 0.85-1.03]; *P* = .006 for trend). Long-term use of low-dose aspirin was significantly associated with reduced risk of lung cancer among nonsmokers (aHR: 5-6 years’ use, 0.94 [95% CI, 0.89-1.00]; 7-8 years’ use, 0.95 [95% CI, 0.89-1.02]; 9 years’ use, 0.89 [95% CI, 0.81-0.98]; *P* = .003 for trend), among smokers with less than 30 pack-years (aHR: 5-6 years’ use, 0.99 [95% CI, 0.92-1.06]; 7-8 years’ use, 0.91 [95% CI, 0.83-0.99]; 9 years’ use, 0.90 [95% CI, 0.80-1.01]; *P* = .03 for trend), and among smokers with more than 30 pack-years (aHR: 5-6 years’ use, 0.97 [95% CI, 0.91-1.04]; 7-8 years’ use, 0.97 [95% CI, 0.90-1.05]; 9 years’ use, 0.89 [95% CI, 0.80-0.99]; *P* = .04 for trend). Long-term use of low-dose aspirin was significantly associated with reduced risk of lung cancer among participants aged 65 years or more (aHR: 5-6 years’ use, 0.95 [95% CI, 0.92-1.00]; 7-8 years’ use, 0.93 [95% CI, 0.89-0.98]; 9 years’ use, 0.87 [95% CI, 0.82-0.93]; *P* < .001 for trend) but not among those aged less than 65 years (aHR: 5-6 years’ use, 0.93 [95% CI, 0.86-1.00]; 7-8 years’ use, 0.99 [95% CI, 0.90-1.08]; 9 years’ use, 0.99 [95% CI, 0.85-1.14]; *P* = .09 for trend). Long-term use of low-dose aspirin was significantly associated with reduced risk of lung cancer among people without diabetes (aHR: 5-6 years’ use, 0.96 [95% CI, 0.92-1.00]; 7-8 years’ use, 0.94 [95% CI, 0.89-0.99]; 9 years’ use, 0.87 [95% CI, 0.81-0.94]; *P* < .001 for trend) but not among those with diabetes (aHR: 5-6 years’ use, 0.97 [95% CI, 0.91-1.04]; 7-8 years’ use, 0.98 [95% CI, 0.91-1.07]; 9 years’ use, 0.95 [95% CI, 0.85-1.06]; *P* = .37 for trend).

The results of the sex- and age-adjusted model are presented in eTable 7 in the Supplement. Sensitivity analyses 1 through 7 for all participants showed trends consistent with the main results (eTables 8 through 17 in the Supplement). Sensitivity analysis 8 showed that 7 to 9 years of low-dose aspirin use among women, obese people, and smokers (<30 pack-years) had a significant association with a lower risk of lung cancer (eTable 18 in the Supplement).

## Discussion

This study showed that low-dose aspirin use for more than 5 years was associated with a significantly lower incidence of lung cancer. Furthermore, except for younger people and participants with diabetes, the risk of incident lung cancer decreased with longer duration of low-dose aspirin use. The effective duration of low-dose aspirin use and proper aspirin dose are clinically important factors to avoid adverse effects of aspirin. A previous study reported that the risk of gastrointestinal bleeding increased with increasing frequency of weekly standard-dose aspirin use, and that the adverse effect was more closely associated with aspirin dose than duration of aspirin use.^24^ To our knowledge, the association between long-term use of low-dose aspirin and the decreased risk of incident lung cancer is a novel finding with implications for a new strategy in lung cancer prevention. In addition, the present study provided evidence for modifying the association of decreased risk by age and history of diabetes, whereas sex, obesity, and smoking history did not modify the association between low-dose aspirin use and the risk of incident lung cancer.

According to previous epidemiologic studies, the association between low-dose aspirin use and risk of lung cancer remains controversial. A case-control study reported that intake of aspirin regularly (≥3 standard [325 mg] tablets/week) for more than 5 years was inversely associated with the risk of lung cancer (odds ratio, 0.36 [95% CI, 0.22-0.58]).^25^ A pooled analysis of randomized trials with 20 years’ follow-up showed that daily aspirin use reduced deaths due to lung cancer (HR, 0.71 [95% CI, 0.58-0.89]).^12^ The beneficial effect increased with the duration of aspirin use and at ages 65 years or older.^12^ By contrast, the benefit was not associated with aspirin dose (75 mg and upward), sex, or smoking.^12^ Although the effect size of our study was quite modest compared with pooled analyses, other results of pooled analyses were similar to ours. The Nurses’ Health Study demonstrated a nonsignificant 16% lower risk of lung cancer for regular (1 or 2 tablets per week) aspirin users among women, whereas the risk of lung cancer increased by 55% among aspirin users who took 15 or more tablets per week.^22^ A nested case-control study also showed 53% increased risk of lung cancer was reported among participants who had been prescribed aspirin of 150 mg or more per day for at least 1 year.^26^ The Women’s Health Study reported no significant association between long-term alternate-day aspirin use (100 mg) and lung cancer risk among women after a median 10 years of follow-up.^27^ A cohort study reported that daily use of adult-strength aspirin (≥325 mg) for more than 5 years was not significantly associated with the risk of lung cancer.^23^ A 32-year prospective cohort study following up 88 084 women from the Nurses’ Health Study and 47 881 men from the Health Professionals Follow-up Study reported no significant association between regular use of aspirin and reduced risk of lung cancer.^28^

The present study revealed that the use of low-dose aspirin was not associated with a decreased risk of incident lung cancer among individuals with diabetes and participants younger than 65 years old. People with diabetes have increased platelet turnover and protein glycosylation,^29^ which lowers COX-1 inhibition and aspirin-mediated acetylation.^30^ This mechanism has been suggested as the cause of aspirin resistance in patients with diabetes. A previous pooled analysis of randomized trials demonstrated that the association of aspirin with reduced risk of death due to lung cancer was stronger with increasing age.^12^ The free fraction of salicylate (aspirin) in plasma increases more with age and also significantly reduces clearance of unbounded salicylate in elderly individuals.^31^ However, the precise biological mechanism for this association with age was not clear.

In the present study, low-dose aspirin use for 7 to 8 years, but not 9 years, among women, obese people, or individuals who smoke less than 30 pack-years was associated with a lower risk of lung cancer. Plausible explanations for this result are the relatively small sample size of the 9-year use group (eTable 18 in the Supplement), or the possibility of aspirin resistance in women, obese people, and smokers caused by the presence of estrogen,^32,33^ inflammation,^34^ and thrombus,^35,36^ respectively.

### Strengths and Limitations

Our study had several strengths. First, this was a large-scale, nationwide, population-based, cohort study, and selection bias was reduced by including the majority of the population, which increased statistical power. In addition, a cohort study tends to have better data quality than a case-control study. Second, this study featured a competing risk analysis that represents the absolute risk in the real-world by considering the competing risk.^37^ Long-term aspirin users had higher mortality rates than nonusers in this study (eTable 4 in the Supplement); therefore, the event of interest (occurrence of lung cancer) could be masked or altered by a competing event, such as death. Third, we conducted several sensitivity analyses. All results showed consistency, which increased the reliability of our results. Fourth, we used the KNHID, which stores data that reflect clinical practice and real-world settings, and therefore the recall bias was reduced when compared with data collected using a questionnaire. Fifth, we used codes from the expanded insurance benefit coverage to define lung cancer. The use of these codes increased the accuracy of lung cancer diagnosis in our study (eTable 2 in the Supplement) because the diagnosis was confirmed by a physician to avail the benefit of expanded insurance coverage.

This study also had some limitations. First, it was a retrospective, nonrandomized, cohort study. However, the KNHID is a large-scale, nationwide, population-based database that provides avoidance of selection bias. Second, the effect size of this study was quite modest. Third, the KNHID database did not permit the distinguishing of cancer subtypes, and we were unable to obtain data to investigate the association between histologic types of lung cancer and aspirin use. Previous studies have reported that chemoprevention was limited, particularly for adenocarcinoma. Fourth, we could not capture the duration of low-dose aspirin use before enrollment. As shown in eTable 5 in the Supplement, those who took low-dose aspirin between 2002 and 2010 were more likely to take low-dose aspirin between 2011 and 2015. Therefore, the duration of low-dose aspirin use in the present study could have been underestimated. Fifth, study results have shown year to year variations by the type of analysis and type of subgroup. Because this study was an observational study using census data, the size and the characteristics of the participants varied according to the years of low-dose aspirin use. We performed adjustments of several confounders, stratification analysis, and competing risk analysis to reduce biases. However, it may be insufficient for clarifying the appropriate duration of aspirin use and subgroups having greater chemopreventive benefit.

## Conclusions

The use of low-dose aspirin for more than 5 years may be associated with a lower risk of lung of cancer among the elderly and among people without diabetes, regardless of sex, obesity, and smoking history. In addition, the longer use of low-dose aspirin and the lower risk of lung cancer were mutually associated, as clarified by several sensitivity analyses. However, given the limitations of the present study, further prospective studies are needed to establish whether a causal association exists between aspirin use and risk of lung cancer.

## Notes

Supplement. **eTable 1.** Characteristics of the Study Participants Included in and Excluded From the Analyses **eTable 2.** Comparison of the Incident Number of Patients With Lung Cancer From the Korea Central Cancer Registry Database and Korea National Health Information Database **eTable 3.** Summary of Low-Dose Aspirin Prescription Information for Doses Prescribed to Eligible Participants Between 2002 and 2010 **eTable 4.** Comparison of Competing Event (Death) Occurrence With Duration of Low-Dose Aspirin Use **eTable 5.** Comparison of Low-Dose Aspirin Use During the Follow-up Periods by Duration of Low-Dose Aspirin Use Between 2002 and 2010 **eTable 6.** Characteristics of Participants Without Lung Cancer and Patients With Lung Cancer **eTable 7.** Age- and Sex-Adjusted HRs for Lung Cancer Associated With Duration of Low-Dose Aspirin Use **eTable 8.** Results of the Sensitivity Analysis (i) **eTable 9.** Result of the Sensitivity Analysis (ii) **eTable 10.** Results of the Sensitivity Analysis (iii) **eTable 11.** Results of the Sensitivity Analysis (iv) **eTable 12.** Results of the Sensitivity Analysis (v)^a^ **eTable 13.** Results of the Sensitivity Analysis (v)^a^ **eTable 14.** Results of the Sensitivity Analysis (v)^a^ **eTable 15.** Results of the Sensitivity Analysis (v)^a^ **eTable 16.** Results of the Sensitivity Analysis (vi) **eTable 17.** Results of the Sensitivity Analysis (vii) **eTable 18.** Results of the Sensitivity Analysis (viii)
