# Error in Byline

**DOI:** 10.1001/jamanetworkopen.2019.2366

**Published:** 2019-03-22

In the Original Investigation titled “Association of Long-term Use of Low-Dose Aspirin as Chemoprevention With Risk of Lung Cancer,”^1^ published March 1, 2019, there was an error in the author byline. The first author’s first name is Shinhee. This article has been corrected.^1^
