# Supplementary material for: Association of Long-term Use of Low-Dose Aspirin as Chemoprevention With Risk of Lung Cancer
Source: JAMA Netw Open. 2019 Mar 1;2(3):e190185. doi: 10.1001/jamanetworkopen.2019.0185 (PMC6484667; doi:10.1001/jamanetworkopen.2019.0185)
Supplement: Supplement. — eTable 1. Characteristics of the Study Participants Included in and Excluded From the Analyses eTable 2. Comparison of the Incident Number of Patients With Lung Cancer From the Korea Central Cancer Registry Database and Korea National Health Information Database eTable 3. Summary of Low-Dose Aspirin Prescription Information for Doses Prescribed to Eligible Participants Between 2002 and 2010 eTable 4. Comparison of Competing Event (Death) Occurrence With Duration of Low-Dose Aspirin Use eTable 5. Comparison of Low-Dose Aspirin Use During the Follow-up Periods by Duration of Low-Dose Aspirin Use Between 2002 and 2010 eTable 6. Characteristics of Participants Without Lung Cancer and Patients With Lung Cancer eTable 7. Age- and Sex-Adjusted HRs for Lung Cancer Associated With Duration of Low-Dose Aspirin Use eTable 8. Results of the Sensitivity Analysis (i) eTable 9. Result of the Sensitivity Analysis (ii) eTable 10. Results of the Sensitivity Analysis (iii) eTable 11. Results of the Sensitivity Analysis (iv) eTable 12. Results of the Sensitivity Analysis (v)a eTable 13. Results of the Sensitivity Analysis (v)a eTable 14. Results of the Sensitivity Analysis (v)a eTable 15. Results of the Sensitivity Analysis (v)a eTable 16. Results of the Sensitivity Analysis (vi) eTable 17. Results of the Sensitivity Analysis (vii) eTable 18. Results of the Sensitivity Analysis (viii) [file jamanetwopen-2-e190185-s001.pdf]

Supplementary Online Content

Ye S, Lee M, Lee D, Ha E-H, Chun EM. Association of long-term use of low-dose aspirin as chemoprevention with risk of lung cancer. *JAMA Netw Open*. 2019;2(3):e190185. doi:10.1001/jamanetworkopen.2019.0185

eTable 1. Characteristics of the Study Participants Included in and Excluded From the Analyses

eTable 2. Comparison of the Incident Number of Patients With Lung Cancer From the Korea Central Cancer Registry Database and Korea National Health Information Database

eTable 3. Summary of Low-Dose Aspirin Prescription Information for Doses Prescribed to Eligible Participants Between 2002 and 2010

eTable 4. Comparison of Competing Event (Death) Occurrence With Duration of Low-Dose Aspirin Use

eTable 5. Comparison of Low-Dose Aspirin Use During the Follow-up Periods by Duration of Low-Dose Aspirin Use Between 2002 and 2010

eTable 6. Characteristics of Participants Without Lung Cancer and Patients With Lung Cancer

eTable 7. Age- and Sex-Adjusted HRs for Lung Cancer Associated With Duration of Low-Dose Aspirin Use

eTable 8. Results of the Sensitivity Analysis (i)

eTable 9. Result of the Sensitivity Analysis (ii)

eTable 10. Results of the Sensitivity Analysis (iii)

eTable 11. Results of the Sensitivity Analysis (iv)

eTable 12. Results of the Sensitivity Analysis (v)[a]

eTable 13. Results of the Sensitivity Analysis (v)[a]

eTable 14. Results of the Sensitivity Analysis (v)[a]

eTable 15. Results of the Sensitivity Analysis (v)[a]

eTable 16. Results of the Sensitivity Analysis (vi)

eTable 17. Results of the Sensitivity Analysis (vii)

eTable 18. Results of the Sensitivity Analysis (viii)

© 2019 Ye S et al. *JAMA Network Open*.

This supplementary material has been provided by the authors to give readers additional information about their work.

© 2019 Ye S et al. *JAMA Network Open*.

eTable 1. Characteristics of the Study Participants Included in and Excluded From the Analyses

Characteristics	Study participants, No. (%)		
	All (n=12,998,062)	Included (n=12,969,400)	Excluded (n=28,662)
**Sex**			
Men	6,342,804 (48.8)	6,324,755 (48.8)	18,049 (63.0)
Women	6,655,258 (51.2)	6,644,645 (51.2)	10,613 (37.0)
**Age, y**			
40-49	4,386,716 (33.8)	4,385,141 (33.8)	1,575 (5.5)
50-59	4,231,534 (32.6)	4,226,072 (32.6)	5,462 (19.1)
60-69	2,659,327 (20.5)	2,649,272 (20.4)	10,055 (35.1)
70-79	1,510,662 (11.6)	1,500,584 (11.6)	10,078 (35.2)
80-84	209,823 (1.6)	208,331 (1.6)	1,492 (5.2)
**Income, ventile**			
1-4	1,902,656 (14.6)	1,898,996 (14.6)	3,660 (12.8)
5-8	1,740,857 (13.4)	1,737,649 (13.4)	3,208 (11.2)
9-12	2,070,752 (15.9)	2,066,195 (15.9)	4,557 (15.9)
13-16	2,830,348 (21.8)	2,823,997 (21.8)	6,351 (22.2)
17-20	4,141,722 (31.9)	4,131,742 (31.9)	9,980 (34.8)
Unknown	311,727 (2.4)	310,821 (2.4)	906 (3.2)
**Residential area**			
Metropolitan	5,732,379 (44.1)	5,720,690 (44.1)	11,689 (40.8)
City	5,745,438 (44.2)	5,733,185 (44.2)	12,253 (42.7)
Rural area	1,520,231 (11.7)	1,515,511 (11.7)	4,720 (16.5)
Unknown	14 (0.0)	14 (0.0)	0 (0.0)

© 2019 Ye S et al. *JAMA Network Open*.

eTable 2. Comparison of the Incident Number of Patients With Lung Cancer From the Korea Central Cancer Registry Database and Korea National Health Information Database[a]

	Incident number reported from KCCRD			Incident number defined by ICD-10 (C33.x and C34.x) with code for EBC in KNHID						Incident number defined by only ICD-10 (C33.x and C34.x) in KNHID					
**Years**	**M1[b]**	**F1[c]**	**T1[d]**	**M2[e]**	**△(M2–M1)**	**F2[f]**	**△(F2–F1)**	**T2[g]**	**△(T2–T1)**	**M3[h]**	**△(M3–M1)**	**F3[i]**	**△(F3–F1)**	**T3[j]**	**△(T3–T1)**
**2011**	15,167	6,586	21,753	15,906	739	7,001	415	22,907	1,154	20,292	5,125	10,055	3,469	30,347	8,594
**2012**	15,367	6,751	22,118	16,014	647	7,311	560	23,325	1,207	20,165	4,798	10,280	3,529	30,445	8,327
**2013**	16,171	7,006	23,177	17,014	843	7,555	549	24,569	1,392	20,692	4,521	10,308	3,302	31,000	7,823
**2014**	16,750	7,277	24,027	17,672	922	7,845	568	25,517	1,490	21,183	4,433	10,572	3,295	31,755	7,728
**2015**	17,015	7,252	24,267	17,883	868	7,810	558	25,693	1,426	21,400	4,385	10,330	3,078	31,730	7,463

Abbreviations: KCCRD, Korea Central Cancer Registry Database; ICD-10, International Classification of Diseases, 10th Revision; EBC, expanding benefit coverage; KNHID, Korean National Health Information Database.

[a] The accuracy of definition of patients with lung cancer in the present study was confirmed by comparing the incident number of patients with lung cancer from the Korea Central Cancer Registry Database (KCCRD) with the Korean National Health Information Database (KNHID).

[b] M1 means incidence number of male patients with lung cancer reported by the KCCRD.

[c] F1 means incidence number of female patients with lung cancer reported by the KCCRD.

[d] T1 means incidence number of total patients with lung cancer reported by the KCCRD.

[e] M2 means incidence number of male patients with lung cancer defined by International Classification of Diseases, 10th Revision (ICD-10) with expanding benefit coverage (EBC), using the KNHID.

[f] F2 means incidence number of female patients with lung cancer defined by ICD-10 with EBC, using the KNHID.

[g] T2 means incidence number of total patients with lung cancer defined by ICD-10 with EBC, using the KNHID.

[h] M3 means incidence number of male patients with lung cancer defined by ICD-10, using the NHID.

[i] F3 means incidence number of female patients with lung cancer defined by ICD-10, using the KNHID.

[j] T3 means incidence number of total patients with lung cancer defined by ICD-10, using the KNHID.

© 2019 Ye S et al. *JAMA Network Open.*

eTable 3. Summary of Low-Dose Aspirin Prescription Information for Doses Prescribed to Eligible Participants Between 2002 and 2010[a]

Prescription Information	Specific dose of the prescribed low-dose aspirin, No. (%)			
	75mg	81mg	100mg	Total
**Number of tablets taken at a time**				
< One tablet	0 (0.0)	138 (0.3)	261,382 (0.4)	261,520 (0.4)
One tablet	1 (100.0)	39,350 (97.7)	68,874,868 (94.7)	68,874,868 (94.7)
> One tablet	0 (0.0)	787 (2.0)	894,172 (1.2)	894,959 (1.2)
Unknown	0 (0.0)	0 (0.0)	2,690,411 (3.7)	2 690,411 (3.7)
**Dose frequency**				
< Once a day	0 (0.0)	0 (0.0)	24,815 (0.0)	24,815 (0.0)
Once a day	1 (100.0)	38,464 (95.5)	68,810,190 (94.6)	68,848,655 (94.6)
> Once a day	0 (0.0)	1,811 (4.5)	3,885,828 (5.3)	3,887,639 (5.3)
**Days prescribed at a visit of a hospital**				
< 30 days	0 (0.0)	9,925 (24.6)	17,994,137 (24.7)	18,004,062 (24.7)
30 days	1 (100.0)	26,837 (66.6)	39,925,264 (54.9)	39,952,102 (54.9)
> 30 days	0 (0.0)	3,513 (8.7)	14,801,432 (20.4)	14,804,945 (20.4)

[a] Prescribing data in the health care utilization database included dose per one tablet, number of tablets taken at a time, frequency of doses per day, and days prescribed at a hospital visit. Eligible study participants were prescribed low-dose aspirin 72,611,109 times between 2002 and 2010. Analysis of this prescription data showed that 94.7% of the prescriptions instructed taking one tablet at a time, and 94.6% of the prescriptions directed taking aspirin once a day. Most of the participants used one low-dose aspirin tablet at a time, one time per day.

© 2019 Ye S et al. *JAMA Network Open.*

eTable 4. Comparison of Competing Event (Death) Occurrence With Duration of Low-Dose Aspirin Use

	Types of follow-up loss, No. (%)		
**Duration of aspirin use, years**	**Censoring (n=12,588,924)**	**Event of interest (Lung cancer, n=63,040)**	**Competing event (Death, n=317,436)**
None	10,729,264 (85.2)	46,309 (73.5)	211,844 (66.7)
1-2	707,280 (5.6)	6,009 (9.5)	37,703 (11.9)
3-4	476,787 (3.8)	4,205 (6.7)	25,953 (8.2)
5-6	347,882 (2.8)	3,207 (5.1)	19,973 (6.3)
7-8	223,875 (1.8)	2,223 (3.5)	14,430 (4.6)
9	103,836 (0.8)	1,087 (1.7)	7,533 (2.4)

© 2019 Ye S et al. *JAMA Network Open*.

eTable 5. Comparison of Low-Dose Aspirin Use During the Follow-up Periods by Duration of Low-Dose Aspirin Use Between 2002 and 2010

	Duration of low-dose aspirin use between 2002 and 2010, years					
**Characteristics**	**None (n=10,987,417)**	**1-2 (n=750,992)**	**3-4 (n=506,945)**	**5-6 (n=371,062)**	**7-8 (n=240,528)**	**9 (n=112,456)**
**Aspirin use during follow-up period, No. (%)**						
No	10,281,267 (93.6)	330,243 (44.0)	139,808 (27.6)	70,415 (19.0)	32,556 (13.5)	6,786 (6.03)
Yes	706,150 (6.4)	420,749 (56.0)	367,137 (72.4)	300,647 (81.0)	207,972 (86.5)	105,670 (94.0)
**Mean aspirin use duration during follow-up period, mean (SD), years**						
	0.2 (0.7)	2.0 (2.1)	2.8 (2.1)	3.2 (2.0)	3.5 (1.9)	4.0 (1.6)
**Duration of aspirin use during follow-up period, No. (%), years**						
0	1,0281,267 (93.6)	330,243 (44.0)	139,808 (27.6)	70,415 (19.0)	32,556 (13.5)	6,786 (6.0)
1-2	406,377 (3.7)	130,681 (17.4)	88,287 (17.4)	61,502 (16.6)	37,332 (15.5)	15,813 (14.1)
3-4	219,004 (2.0)	101,173 (13.5)	81,365 (16.1)	61,811 (16.7)	40,163 (16.7)	18,403 (16.4)
5	80,769 (0.7)	188,895 (25.2)	197,485 (39.0)	177,334 (47.8)	130,477 (54.3)	71,454 (63.5)

Abbreviation: SD, Standard Deviation.

© 2019 Ye S et al. *JAMA Network Open*.

eTable 6. Characteristics of Participants Without Lung Cancer and Patients With Lung Cancer[a]

	Participants, No. (%)	
**Characteristics**	**Participants without lung cancer (n=12,906,360)**	**Patients with lung cancer (n=63,040)**
**Sex**		
Men	6,279,599 (48.7)	45,156 (71.6)
Women	6,626,761 (51.3)	17,884 (28.4)
**Age, y**		
40-49	4,381,879 (34.0)	3,262 (5.2)
50-59	4,214,378 (32.7)	11,694 (18.6)
60-69	2,627,527 (20.4)	21,745 (34.5)
70-79	1,477,852 (11.5)	22,732 (36.1)
80-84	204,724 (1.6)	3,607 (5.7)
**Income, ventile[b]**		
1-4	1,889,610 (14.6)	9,386 (14.9)
5-8	1,729,759 (13.4)	7,890 (12.5)
9-12	2,056,169 (15.9)	10,026 (15.9)
13-16	2,810,010 (21.8)	13,987 (22.2)
17-20	4,111,654 (31.9)	20,088 (31.9)
Unknown	309,158 (2.4)	1,663 (2.6)
**Residential area[c]**		
Metropolitan	5,696,002 (44.1)	24,688 (39.2)
Urban	5,706,392 (44.2)	26,793 (42.5)
Rural area	1,503,952 (11.7)	11,559 (18.3)
Unknown	14 (0)	0 (0)
**Smoking status**		
Non-smoker	8,301,701 (64.3)	27,009 (42.8)
Past smoker	2,000,856 (15.5)	12,972 (20.6)
Current smoker	2,603,803 (20.2)	23,059 (36.6)
**Pack-years of smoking, pack-years[d]**		
Non-smoker	8,306,797 (64.4)	27,041 (42.9)
<30	3,382,125 (26.2)	16,257 (25.8)
≥30	1,087,164 (8.4)	18,980 (30.1)
Unknown	130,274 (1.0)	762 (1.2)
**Alcohol consumption, units/week[e]**		
No alcohol consumption	7,558,281 (58.6)	36454(57.8)
1-7	2,238,410 (17.3)	9034(14.3)
8-14	1,129,724 (8.8)	5592(8.9)
≥15	1845924(14.3)	11226(17.8)
Unknown	134021(1)	734(1.2)
**Moderate or vigorous exercise, times/week[f]**		
No exercise	6,408,135 (49.7)	35,775 (56.8)
1-4	3,901,598 (30.2)	14,383 (22.8)
≥5	2,517,732 (19.5)	12,384 (19.6)
Unknown	78,895 (0.6)	498 (0.8)
**BMI, kg/m^2**		

Characteristics	Participants, No. (%)	
	Participants without lung cancer (n=12,906,360)	Patients with lung cancer (n=63,040)
<18.5	295,966 (2.3)	2,843 (4.5)
18.5-22.9	4,650,492 (36.0)	25,082 (39.8)
23-24.9	3,404,699 (26.4)	16,273 (25.8)
25-29.9	4,100,405 (31.8)	17,304 (27.5)
≥30	451,033 (3.5)	1,510 (2.4)
Unknown	3,765 (0.0)	28.0 (0.0)
**History of DM[g]**		
No	11,580,418 (89.7)	53,080 (84.2)
Yes	1,325,942 (10.3)	9,960 (15.8)
**Family history of cancer**		
No	7,803,760 (60.5)	41,881 (66.4)
Yes	2,089,688 (16.2)	9,283 (14.7)
Unknown	3,012,912 (23.3)	11,876 (18.8)
**CCI score[h]**		
0	4,001,596 (31.0)	2,871 (4.6)
1	3,837,603 (29.7)	9,482 (15.0)
2	2,416,615 (18.7)	16,380 (26.0)
3	1,511,554 (11.7)	18,322 (29.1)
≥4	1,138,992 (8.8)	15,985 (25.4)

Abbreviations: BMI, body mass index; DM, diabetes mellitus; CCI, Charlson comorbidity index; SD, standard deviation; ICD-10, International Classification of Diseases, 10th revision.

[a] Patients with lung cancer were defined as those who had International Classification of Diseases, 10th revision (ICD-10) codes C33.x (malignant neoplasm of trachea) or C34.x (malignant neoplasm of bronchus and lung) in principal or first additional diagnosis with code for expanding benefit coverage during follow up between January 1, 2011 and December 31, 2015.

[b] The incomes were grouped into ventiles based on the population size of each national health insurance-determined premium level.

[c] Residential area was classified into metropolitan, city, and rural areas, based on residential addresses of participants.

[d] If participants responded as past smoker or current smoker on the questionnaire of national health screening, pack-years of smoking for those people were calculated by multiplying years of cigarettes smoked and number of cigarettes typically smoked in a day; those who smoked 0 pack-years of smoking were reclassified into non-smokers.

[e] Quantity of alcohol consumption per week was calculated based on the questionnaire of national health screening, and types of alcohol were not considered.

[f] Frequency of moderate or vigorous exercise per week was calculated based on the national health screening questionnaire.

[g] History of DM defined by fasting serum glucose level (≥126mg/dL) or type 1 and 2 diabetes mellitus diagnosis (ICD-10 codes: E10.x and E11.x).

[h] CCI score calculated using ICD-10 codes from the health care utilization database.

© 2019 Ye S et al. *JAMA Network Open*.

eTable 7. Age- and Sex-Adjusted HRs for Lung Cancer Associated With Duration of Low-Dose Aspirin Use

Duration of aspirin use, years	Cases	Person-years	Age- and sex-adjusted model	
			HR[a] (95% CI)	*P*
**All participants**				
None	46,309	54,179,622.6	Reference	
1-2	6,009	3,644,691.1	1.00 (0.97-1.03)	0.86
3-4	4,205	2460400.8	0.96 (0.93-1.00)	0.03
5-6	3,207	1798642.4	0.93 (0.90-0.96)	<0.001
7-8	2,223	1162068.0	0.90 (0.86-0.94)	<0.001
9	1,087	541833.2	0.83 (0.79-0.89)	<0.001
P for trend[b]				<0.001
**Sex**				
**Men**				
None	32,833	26,194,276.6	Reference	
1-2	4,395	1,769,922.9	1.01 (0.98-1.04)	0.68
3-4	3,060	1,214,527.9	0.96 (0.92-1.00)	0.03
5-6	2,365	888,871.4	0.92 (0.88-0.96)	<0.001
7-8	1,676	575,953.3	0.89 (0.85-0.94)	<0.001
9	827	280,069.5	0.80 (0.75-0.86)	<0.001
P for trend[b]				<0.001
**Women**				
None	13,476	27,985,497.9	Reference	
1-2	1,614	1,874,758.4	1.01 (0.95-1.06)	0.85
3-4	1,145	1,245,876.5	1.01 (0.95-1.08)	0.65
5-6	842	909,775.5	0.97 (0.90-1.04)	0.40
7-8	547	586,115.6	0.92 (0.85-1.01)	0.07
9	260	261,763.9	0.93 (0.82-1.05)	0.23
P for trend[b]				0.07
**Age**				
**<65 years**				
None	20,267	44,900,562.9	Reference	
1-2	1,621	2,047,163.0	1.04 (0.98-1.09)	0.17

© 2019 Ye S et al. *JAMA Network Open.*

Duration of aspirin use, years	Cases	Person-years	Age- and sex-adjusted model	
			HR[a] (95% CI)	*P*
3-4	1,018	1,285,327.4	0.96 (0.90-1.02)	0.23
5-6	696	847,988.8	0.93 (0.86-1.00)	0.05
7-8	442	479,320.2	0.97 (0.89-1.07)	0.59
9	187	189,995.5	0.96 (0.83-1.11)	0.58
P for trend[b]				0.08
**≥65 years**				
None	26,042	9,279,161.4	Reference	
1-2	4,388	1,597,527.2	0.97 (0.94-1.00)	0.04
3-4	3,187	1,175,085.1	0.95 (0.91-0.98)	0.004
5-6	2,511	950,658.1	0.91 (0.87-0.95)	<0.001
7-8	1,781	682,751.1	0.87 (0.83-0.91)	<0.001
9	900	351,838.2	0.81 (0.76-0.86)	<0.001
P for trend[b]				<0.001
**BMI**				
**<25 kg/m^2**				
None	33,978	36,344,390.8	Reference	
1-2	3,805	1,952,846.0	0.99 (0.96-1.02)	0.52
3-4	2,583	1,240,435.2	0.98 (0.94-1.02)	0.28
5-6	1,888	880,858.0	0.93 (0.89-0.97)	0.002
7-8	1,341	557,115.4	0.94 (0.89-0.99)	0.03
9	603	257,790.3	0.81 (0.75-0.88)	<0.001
P for trend[b]				<0.001
**≥25 kg/m^2**				
None	12,331	17,835,222.7	Reference	
1-2	2,204	1,691,837.8	1.08 (1.03-1.13)	0.001
3-4	1,622	1,219,975.3	1.02 (0.97-1.08)	0.37
5-6	1,319	917,791.1	1.01 (0.96-1.07)	0.67
7-8	882	604,954.3	0.92 (0.86-0.99)	0.02
9	484	284,044.8	0.95 (0.87-1.04)	0.29
P for trend[b]				0.19

© 2019 Ye S et al. *JAMA Network Open.*

Duration of aspirin use, years	Cases	Person-years	Age- and sex-adjusted model	
			HR[a] (95% CI)	*P*
**History of DM**				
**No**				
None	40,983	50,162,057.3	Reference	
1-2	4,504	2,795,007.5	0.98 (0.95-1.01)	0.28
3-4	3,018	1,827,624.4	0.94 (0.90-0.97)	0.001
5-6	2,280	1,311,315.0	0.91 (0.87-0.95)	<0.001
7-8	1,545	835,252.2	0.87 (0.83-0.92)	<0.001
9	750	389,223.5	0.81 (0.75-0.87)	<0.001
P for trend[b]				<0.001
**Yes**				
None	5,326	4,017,546.6	Reference	
1-2	1,505	849,676.5	1.02 (0.97-1.09)	0.41
3-4	1,187	632,777.9	1.03 (0.96-1.09)	0.41
5-6	927	487,327.9	0.96 (0.90-1.04)	0.32
7-8	678	326,814.6	0.96 (0.88-1.04)	0.29
9	337	152,611.1	0.91 (0.81-1.01)	0.09
P for trend[b]				0.09
**Pack-years of smoking**				
**Non-smoker**				
None	19,811	34,693,401.0	Reference	
1-2	2,579	2,410,135.5	1.01 (0.97-1.06)	0.54
3-4	1,842	1,631,207.3	1.01 (0.96-1.06)	0.80
5-6	1,377	1,205,045.8	0.96 (0.9-1.01)	0.11
7-8	978	786,832.7	0.96 (0.9-1.03)	0.24
9	454	362,428.4	0.89 (0.81-0.98)	0.01
P for trend[b]				0.01
**<30 pack-years**				
None	12,129	14,678,119.9	Reference	
1-2	1,478	785,404.0	1.01 (0.96-1.07)	0.72
3-4	1,021	526,046.8	0.96 (0.90-1.02)	0.18

Duration of aspirin use, years	Cases	Person-years	Age- and sex-adjusted model	
			HR[a] (95% CI)	*P*
5-6	803	372,428.5	0.94 (0.87-1.01)	0.09
7-8	537	235,094.9	0.85 (0.78-0.93)	<0.001
9	289	111,847.2	0.83 (0.74-0.94)	0.002
P for trend[b]				<0.001
**≥30 pack-years**				
None	13,789	4,252,976.7	Reference	
1-2	1,890	416,019.7	0.98 (0.93-1.03)	0.45
3-4	1,295	280,450.4	0.95 (0.90-1.00)	0.07
5-6	993	205,009.8	0.93 (0.87-0.99)	0.03
7-8	681	129,468.9	0.92 (0.85-0.99)	0.03
9	332	62,130.6	0.83 (0.74-0.92)	0.001
P for trend[b]				<0.001

Abbreviations: HR, hazard ratio; CI, confidence interval.
[a] HRs were calculated by competing risk analysis (Fine and Gray model) and were adjusted for age and sex.
[b] *P* value for trend across ordered categories.

© 2019 Ye S et al. *JAMA Network Open*.

eTable 8. Results of the Sensitivity Analysis (i)[a]

Duration of aspirin use, years	Cases	Person-years	Fully adjusted model	
			HR[b] (95% CI)	*P*
None	46,309	54,179,622.6	Reference	
1-2	6,009	3,644,691.1	1.00 (0.97-1.02)	0.74
3-4	4,205	2,460,400.8	0.98 (0.95-1.01)	0.24
5-6	3,207	1,798,642.4	0.96 (0.92-0.99)	0.02
7-8	2,223	1,162,068.0	0.94 (0.90-0.99)	0.01
9	1,087	541,833.2	0.89 (0.84-0.94)	<0.001
P for trend[c]				<0.001

Abbreviations: HR, hazard ratio; CI, confidence interval; BMI, body mass index; DM, diabetes mellitus; CCI, Charlson Comorbidity Index.

[a] A conventional survival analysis (Cox proportional hazards model) was conducted to compare the results of the competing risk analysis because previous cohort studies on the association between aspirin use and lung cancer were conducted via conventional survival analysis, wherein death was considered a censoring event.

[b] HRs were calculated by conventional survival analysis (Cox proportional hazards model), and were adjusted for age, sex, income, residential area, pack-years of smoking, quantity of alcohol consumed, frequency of moderate or vigorous exercise per week, body mass index (BMI), history of diabetes mellitus (DM), family history of cancer, and Charlson Comorbidity Index (CCI) score.

[c] *P* value for trend across ordered categories.

eTable 9. Results of the Sensitivity Analysis (ii)[a]

Duration of aspirin use, years	Cases	Person-years	Fully adjusted model	
			HR[b] (95% CI)	*P*
None	17,993	9,644,240.0	Reference	
1-2	6,009	3,644,691.0	0.97 (0.94-1.00)	0.04
3-4	4,205	2,460,401.0	0.96 (0.93-0.99)	0.02
5-6	3,207	1,798,642.0	0.94 (0.91-0.98)	0.003
7-8	2,223	1,162,068.0	0.94 (0.90-0.98)	0.006
9	1,087	541,833.2	0.89 (0.84-0.95)	<0.001
P for trend[c]				<0.001

Abbreviations: PS, propensity score; HR, hazard ratio; CI, confidence interval; BMI, body mass index; DM, diabetes mellitus; CCI, Charlson Comorbidity Index.

[a] We compared lung cancer risk between aspirin users and non-users in a 1:1 propensity score (PS)-matched cohort study as follows. First, we created a PS for aspirin users and non-users using variables of sex and baseline age. Second, we performed a 1:1 case-control (aspirin users and non-users) match on the PS, using the greedy matching macro (http://www2.sas.com/proceedings/sugi29/165-29.pdf). Third, in the 1:1 PS-matched cohort, we analyzed the association between the duration of low-dose aspirin use and lung cancer risk using competing risk analysis.

[b] HRs were calculated by competing risk analysis (Fine and Gray model), and were adjusted for age, sex, income, residential area, pack-years of smoking, quantity of alcohol consumed, frequency of moderate or vigorous exercise per week, body mass index (BMI), history of diabetes mellitus (DM), family history of cancer, and Charlson Comorbidity Index (CCI) score.

[c] *P* value for trend across ordered categories.

© 2019 Ye S et al. *JAMA Network Open*.

eTable 10. Results of the Sensitivity Analysis (iii)[a]

Duration of aspirin use, years	Cases	Person-years	Fully adjusted model	
			HR[b] (95% CI)	*P*
None	43,570	50,712,059.3	Reference	
1-2	6,136	3,778,823.0	0.94 (0.91-0.97)	<0.001
3-4	4,260	2,428,144.0	0.94 (0.91-0.97)	<0.001
5-6	3,438	2,041,258.4	0.87 (0.84-0.91)	<0.001
7-8	2,713	1,762,937.0	0.80 (0.77-0.83)	<0.001
9	2,923	3,064,077.1	0.46 (0.45-0.48)	<0.001
P for trend[c]				<0.001

Abbreviations: HR, hazard ratio; CI, confidence interval; BMI, body mass index; DM, diabetes mellitus; CCI, Charlson Comorbidity Index.

[a] Because some participants were taking aspirin during the follow-up period (Supplement eTable 5), we calculated the duration of aspirin use from 2002 to the years in which the event occurred or follow-up was censored. Then, we analyzed the association between the incident lung cancer and the duration of aspirin use from 2002 to the years in which the event occurred or the follow-up was censored.

[b] HRs were calculated by competing risk analysis (Fine and Gray model), and were adjusted for age, sex, income, residential area, pack-years of smoking, quantity of alcohol consumed, frequency of moderate or vigorous exercise per week, body mass index (BMI), history of diabetes mellitus (DM), family history of cancer, and Charlson Comorbidity Index (CCI) score.

[c] *P* value for trend across ordered categories.

© 2019 Ye S et al. *JAMA Network Open.*

eTable 11. Results of the Sensitivity Analysis (iv)[a]

Duration of aspirin use, years	Cases	Person-years	Fully adjusted model without family history of cancer	
			HR[b] (95% CI)	*P*
None	46,309	54,179,622.6	Reference	
1-2	6,009	3,644,691.1	0.99 (0.97-1.02)	0.60
3-4	4,205	2,460,400.8	0.98 (0.95-1.01)	0.21
5-6	3,207	1,798,642.4	0.96 (0.92-0.99)	0.02
7-8	2,223	1,162,068.0	0.94 (0.90-0.99)	0.009
9	1,087	541,833.2	0.89 (0.84-0.94)	<0.001
P for trend[c]				<0.001

Abbreviations: HR, hazard ratio; CI, confidence interval; BMI, body mass index; DM, diabetes mellitus; CCI, Charlson Comorbidity Index.
[a] Because 20% of family history data were missing, we also conducted an analysis in the fully adjusted model, after excluding the family history variable.
[b] HRs were calculated by competing risk analysis (Fine and Gray model), and were adjusted for age, sex, income, residential area, pack-years of smoking, quantity of alcohol consumed, frequency of moderate or vigorous exercise per week, body mass index (BMI), history of diabetes mellitus (DM), and Charlson Comorbidity Index (CCI) score.
[c] *P* value for trend across ordered categories.

eTable 12. Results of the Sensitivity Analysis (v)[a]

Duration of aspirin use, years	Cases	Person-years	Fully adjusted model	
			HR[b] (95% CI)	*P*
None	47,515	54,904,980.3	Reference	
1-2	5,675	3,391,869.8	1.00 (0.97-1.03)	0.86
3-4	3,893	2,322,262.2	0.95 (0.92-0.99)	0.005
5-6	3,062	1,682,123.4	0.97 (0.94-1.01)	0.12
7-8	1,983	1,042,143.9	0.93 (0.89-0.97)	0.001
9	912	444,077.6	0.90 (0.84-0.96)	0.001
P for trend[c]				<0.001

Abbreviations: HR, hazard ratio; CI, confidence interval; BMI, body mass index; DM, diabetes mellitus; CCI, Charlson Comorbidity Index.

[a] Duration of low-dose (≤100mg) aspirin use between January 1, 2002 and December 31, 2010 was calculated. People who were prescribed low-dose aspirin for greater than or equal to 156 days during a year, were defined as low-dose aspirin users in the year.

[b] HRs were calculated by competing risk analysis (Fine and Gray model) , and were adjusted for age, sex, income, residential area, pack-years of smoking, quantity of alcohol consumed, frequency of moderate or vigorous exercise per week, body mass index (BMI), history of diabetes mellitus (DM), family history of cancer, and Charlson Comorbidity Index (CCI) score.

[c] *P* value for trend across ordered categories.

© 2019 Ye S et al. *JAMA Network Open*.

eTable 13. Results of the Sensitivity Analysis (v)[a]

Duration of aspirin use, years	Cases	Person-years	Fully adjusted model	
			HR[b] (95% CI)	*P*
None	48,431	55,462,181.0	Reference	
1-2	5,444	3,258,297.1	0.99 (0.96-1.02)	0.41
3-4	3,758	2,215,618.5	0.96 (0.93-0.99)	0.01
5-6	2,900	1,565,049.2	0.98 (0.94-1.02)	0.28
7-8	1,773	926,074.8	0.92 (0.88-0.97)	0.001
9	734	360,149.9	0.88 (0.82-0.95)	0.001
P for trend[c]				<0.001

Abbreviations: HR, hazard ratio; CI, confidence interval; BMI, body mass index; DM, diabetes mellitus; CCI, Charlson Comorbidity Index.

[a] Duration of low-dose (≤100mg) aspirin use between January 1, 2002 and December 31, 2010 was calculated. People who were prescribed low-dose aspirin for greater than or equal to 208 days during a year, were defined as low-dose aspirin users in the year.

[b] HRs were calculated by competing risk analysis (Fine and Gray model), and were adjusted for age, sex, income, residential area, pack-years of smoking, quantity of alcohol consumed, frequency of moderate or vigorous exercise per week, body mass index (BMI), history of diabetes mellitus (DM), family history of cancer, and Charlson Comorbidity Index (CCI) score.

[c] *P* value for trend across ordered categories.

© 2019 Ye S et al. *JAMA Network Open*.

eTable 14. Results of the Sensitivity Analysis (v)[a]

Duration of aspirin use, years	Cases	Person-years	Fully adjusted model	
			HR[b] (95% CI)	*P*
None	49,481	56,143,072.5	Reference	
1-2	5,306	3,133,870.2	1.00 (0.97-1.03)	0.84
3-4	3,538	2,058,736.0	0.96 (0.93-0.99)	0.02
5-6	2,612	1,400,136.8	0.97 (0.93-1.01)	0.17
7-8	1,566	786,875.3	0.94 (0.89-0.99)	0.02
9	537	264,585.6	0.87 (0.80-0.95)	0.001
P for trend[c]				<0.001

Abbreviations: HR, hazard ratio; CI, confidence interval; BMI, body mass index; DM, diabetes mellitus; CCI, Charlson Comorbidity Index.

[a] Duration of low-dose (≤100mg) aspirin use between January 1, 2002 and December 31, 2010 was calculated. People who were prescribed low-dose aspirin for greater than or equal to 260 days during a year, were defined as low-dose aspirin users in the year.

[b] HRs were calculated by competing risk analysis (Fine and Gray model) , and were adjusted for age, sex, income, residential area, pack-years of smoking, quantity of alcohol consumed, frequency of moderate or vigorous exercise per week, body mass index (BMI), history of diabetes mellitus (DM), family history of cancer, and Charlson Comorbidity Index (CCI) score.

[c] *P* value for trend across ordered categories.

eTable 15. Results of the Sensitivity Analysis (v)[a]

Duration of aspirin use, years	Cases	Person-years	Fully adjusted model	
			HR[b] (95% CI)	*P*
<0.5	47,109	54,664,805.1	Reference	
0.5-0.9	2,354	1,431,347.6	0.99 (0.95-1.04)	0.77
1-2.9	5,315	3,201,306.7	0.98 (0.95-1.01)	0.19
3-4.9	3,684	2,123,209.5	0.96 (0.93-0.99)	0.02
5-6.9	2,582	1,387,623.6	0.96 (0.92-1.00)	0.03
7-8.9	1,802	887,013.1	0.91 (0.87-0.96)	<0.001
≥ 9	194	92,047.6	0.88 (0.77-1.01)	0.08
P for trend[c]				<0.001

Abbreviations: HR, hazard ratio; CI, confidence interval; BMI, body mass index; DM, diabetes mellitus; CCI, Charlson Comorbidity Index.

[a] The total days of low-dose aspirin prescribed between January 2002 and December 2010 was used for the exposure.

[b] HRs were calculated by competing risk analysis (Fine and Gray model), and were adjusted for age, sex, income, residential area, pack-years of smoking, quantity of alcohol consumed, frequency of moderate or vigorous exercise per week, body mass index (BMI), history of diabetes mellitus (DM), family history of cancer, and Charlson Comorbidity Index (CCI) score.

[c] *P* value for trend across ordered categories.

© 2019 Ye S et al. *JAMA Network Open*.

eTable 16. Results of the Sensitivity Analysis (vi)[a]

Duration of aspirin use, years	Cases	Person-years	Fully adjusted model	
			HR[b] (95% CI)	*P*
None	46,309	54,179,622.6	Reference	
1-2	6,009	3,644,691.1	1.01 (0.98-1.04)	0.49
3-4	4,205	2,460,400.8	1.00 (0.97-1.03)	0.96
5-6	3,207	1,798,642.4	0.98 (0.94-1.02)	0.24
7-8	2,223	1,162,068.0	0.97 (0.92-1.01)	0.11
9	1,087	541,833.2	0.91 (0.86-0.97)	0.004
P for trend[c]				0.006

Abbreviations: HR, hazard ratio; CI, confidence interval; BMI, body mass index; DM, diabetes mellitus; CCI, Charlson Comorbidity Index.
[a] Smoking status is also a strong risk factor of incident lung cancer. Therefore, in the fully adjusted model, we used smoking status, instead of pack-years of smoking for the adjustment. Smoking status was categorized into non-smoker, past smoker or current smoker based on the national health screening questionnaire.
[b] HRs were calculated by competing risk analysis (Fine and Gray model), and were adjusted for age, sex, income, residential area, smoking status, quantity of alcohol consumed, frequency of moderate or vigorous exercise per week, body mass index (BMI), history of diabetes mellitus (DM), family history of cancer, and Charlson Comorbidity Index (CCI) score.
[c] *P* value for trend across ordered categories.

© 2019 Ye S et al. *JAMA Network Open.*

eTable 17. Results of the Sensitivity Analysis (vii)[a]

Duration of aspirin use, years	Cases	Person-years	Fully adjusted model	
			HR[b] (95% CI)	*P*
None	37,993	54,175,588.6	Reference	
1-2	4,912	3,644,130.4	1.00 (0.97-1.04)	0.77
3-4	3,458	2,460,032.2	1.00 (0.96-1.03)	0.87
5-6	2,627	1,798,366.8	0.97 (0.93-1.01)	0.16
7-8	1,781	1,161,838.8	0.94 (0.89-0.98)	0.009
9	896	541,739.7	0.91 (0.85-0.97)	0.005
P for trend[c]				<0.001

Abbreviations: HR, hazard ratio; CI, confidence interval; BMI, body mass index; DM, diabetes mellitus; CCI, Charlson Comorbidity Index.

[a] Short follow-up time (5 years) can cause a reverse causation or some related bias. Therefore, we analyzed the fully adjusted model after excluding cases (patients with lung cancer) that occurred during the first year of follow-up.

[b] HRs were calculated by competing risk analysis (Fine and Gray model), and were adjusted for age, sex, income, residential area, pack-years of smoking, quantity of alcohol consumed, frequency of moderate or vigorous exercise per week, body mass index (BMI), history of diabetes mellitus (DM), family history of cancer, and Charlson Comorbidity Index (CCI) score.

[c] *P* value for trend across ordered categories.

© 2019 Ye S et al. *JAMA Network Open*.

eTable 18. Results of the Sensitivity Analysis (viii)[a]

Duration of aspirin use, years	Cases	Person-years	Fully adjusted model	
			HR[b] (95% CI)	*P*
**All participants**				
None	46,309	54,179,622.6	Reference	
1-2	6,009	3,644,691.1	0.99 (0.97-1.02)	0.61
3-4	4,205	2,460,400.8	0.98 (0.95-1.01)	0.22
5-6	3,207	1,798,642.4	0.96 (0.92-0.99)	0.02
7-9	3,310	1,703,901.0	0.93 (0.89-0.96)	<0.001
P for trend[c]				<0.001
**Sex**				
**Men**				
None	32,833	26,194,276.6	Reference	
1-2	4,395	1,769,922.9	1.01 (0.98-1.04)	0.65
3-4	3,060	1,214,527.9	0.98 (0.95-1.02)	0.40
5-6	2,365	888,871.4	0.97 (0.93-1.01)	0.12
7-9	2,503	856,022.8	0.93 (0.89-0.97)	<0.001
P for trend[c]				<0.001
**Women**				
None	13,476	27,985,497.9	Reference	
1-2	1,614	1,874,758.4	0.98 (0.93-1.04)	0.49
3-4	1,145	1,245,876.5	1.00 (0.94-1.06)	0.89
5-6	842	909,775.5	0.96 (0.89-1.03)	0.21
7-9	807	847,879.5	0.91 (0.85-0.98)	0.01
P for trend[c]				0.02
**Age**				
**<65 years**				
None	20,267	44,900,562.9	Reference	
1-2	1,621	2,047,163.0	1.00 (0.95-1.06)	0.95
3-4	1,018	1,285,327.4	0.95 (0.89-1.01)	0.13
5-6	696	847,988.8	0.93 (0.86-1.00)	0.05
7-9	629	669,315.7	0.99 (0.91-1.07)	0.74
P for trend[c]				0.08

© 2019 Ye S et al. *JAMA Network Open.*

Duration of aspirin use, years	Cases	Person-years	Fully adjusted model	
			HR[b] (95% CI)	*P*
**≥65 years**				
None	26,042	9,279,161.4	Reference	
1-2	4,388	1,597,527.2	0.98 (0.95-1.01)	0.15
3-4	3,187	1,175,085.1	0.98 (0.94-1.01)	0.23
5-6	2,511	950,658.1	0.96 (0.92-1.00)	0.03
7-9	2,681	1,034,589.3	0.91 (0.87-0.95)	<0.001
P for trend[c]				<0.001
**BMI**				
**<25 kg/m²**				
None	33,978	36,344,390.8	Reference	
1-2	3,805	1,952,846.0	0.96 (0.93-0.99)	0.01
3-4	2,583	1,240,435.2	0.96 (0.92-1.00)	0.04
5-6	1,888	880,858.0	0.92 (0.88-0.97)	<0.001
7-9	1,944	814,905.7	0.91 (0.87-0.95)	<0.001
P for trend[c]				<0.001
**≥25 kg/m²**				
None	12,331	17,835,222.7	Reference	
1-2	2,204	1,691,837.8	1.03 (0.99-1.08)	0.19
3-4	1,622	1,219,975.3	0.98 (0.93-1.04)	0.53
5-6	1,319	917,791.1	0.98 (0.92-1.04)	0.47
7-9	1,366	888,999.1	0.91 (0.86-0.97)	<0.001
P for trend[c]				0.006
**History of DM**				
**No**				
None	40,983	50,162,057.3	Reference	
1-2	4,504	2,795,007.5	0.99 (0.96-1.02)	0.62
3-4	3,018	1,827,624.4	0.97 (0.94-1.01)	0.13
5-6	2,280	1,311,315.0	0.96 (0.92-1.00)	0.06
7-9	2,295	1,224,475.7	0.92 (0.88-0.96)	<0.001
P for trend[c]				<0.001

© 2019 Ye S et al. *JAMA Network Open*.

Duration of aspirin use, years	Cases	Person-years	Fully adjusted model	
			HR[b] (95% CI)	*P*
**Yes**				
None	5,326	4,017,546.6	Reference	
1-2	1,505	849,676.5	1.00 (0.95-1.06)	0.92
3-4	1,187	632,777.9	1.02 (0.96-1.09)	0.55
5-6	927	487,327.9	0.97 (0.91-1.04)	0.44
7-9	1,015	479,425.7	0.97 (0.91-1.04)	0.41
P for trend[c]				0.41
**Pack-years of smoking**				
**Non-smoker**				
None	19,811	34,693,401.0	Reference	
1-2	2,579	2,410,135.5	0.99 (0.95-1.03)	0.57
3-4	1,842	1,631,207.3	0.99 (0.94-1.04)	0.67
5-6	1,377	1,205,045.8	0.94 (0.89-1.00)	0.05
7-9	1,432	1,149,261.1	0.93 (0.88-0.99)	0.01
P for trend[c]				0.005
**<30 pack-years**				
None	12,129	14,678,119.9	Reference	
1-2	1,478	785,404.0	1.03 (0.97-1.08)	0.38
3-4	1,021	526,046.8	0.99 (0.93-1.06)	0.76
5-6	803	372,428.5	0.99 (0.92-1.06)	0.71
7-9	826	346,942.1	0.91 (0.84-0.98)	0.01
P for trend[c]				0.04
**≥30 pack-years**				
None	13,789	4,252,976.7	Reference	
1-2	1,890	416,019.7	1.00 (0.95-1.05)	0.90
3-4	1,295	280,450.4	0.98 (0.92-1.04)	0.46
5-6	993	205,009.8	0.97 (0.91-1.04)	0.43
7-9	1,013	191,599.5	0.94 (0.88-1.01)	0.07
P for trend[c]				0.06

Abbreviations: HR, hazard ratio; CI, confidence interval.
[a] Low-dose aspirin use in women, <30 pack-years smokers or obese subjects for 7-8 years was associated with a lower risk of lung cancer but not at 9 years. A possible explanation for this result is the relatively small sample size of 9-year use

group; thus we conducted a subgroup analysis with a combined 7-9 years use group.
[b] HRs were calculated by competing risk analysis (Fine and Gray model) and were adjusted for age, sex, income, residential area, pack-years of smoking, quantity of alcohol consumed, frequency of moderate or vigorous exercise per week, body mass index (BMI), history of diabetes mellitus (DM), family history of cancer, and Charlson Comorbidity Index (CCI) score. In the stratified analysis by the sex, BMI, history of DM, and pack-years of smoking, each stratification variable was not included in the adjustment variable.
[c] *P* value for trend across ordered categories.

© 2019 Ye S et al. *JAMA Network Open*.
